# Dynamics of CXC group chemokine platelet factor 4 (PF4) plasma levels in non-small cell lung cancer (NSCLC)

**DOI:** 10.1186/1479-5876-10-S3-P9

**Published:** 2012-11-28

**Authors:** Artjoms Spaks, Inta Jaunalksne, Simona Donina, Ainis Pirtnieks, Jelena Grusina-Ujumaza, Irina Spaka, Aurika Babjoniseva, Dainis Krievins

**Affiliations:** 1 Dept. of Thoracic Surgery, Pauls Stradins Clinical University Hospital, Riga, Latvia; 2 Dept. of Clinical Immunology, Pauls Stradins Clinical University Hospital, Riga, Latvia; 3 Dept. of Clinical Immunology, Riga Eastern Clinical University Hospital, Riga, Latvia; 4 Faculty of Biology, University of Latvia, Riga, Latvia; 5 Dept. of Respiratory Medicine, Pauls Stradins Clinical University Hospital, Riga, Latvia; 6 Dept. of Vascular Surgery, Pauls Stradins Clinical University Hospital, Riga, Latvia

## Introduction

CXC chemokines display pleiotropic effects participating not only in inflammation, but regulating angiogenesis and metastatic spread in cancer. Platelet factor 4 (PF4) is a 70-amino acid protein belonging to the CXC chemokine family. PF4 is also known as CXCL4. This chemokine is released from alpha-granules of activated platelets and binds with high affinity to heparin-like molecules promoting coagulation.

Megakaryocytes respond to the presence of tumors by increasing their number in the bone marrow accompanied by increase in the number of platelets in circulation, causing changes in chemokine balance.

## Aim

An attempt was made to get new information about PF4 production in NSCLC and clarify clinical significance of measuring PF4 level and platelet count.

## Patients and methods

Peripheral blood samples were collected from patients with histologically proven early (n=32) and advanced (n=12) stage NSCLC, and healthy individuals (n=12). Complete blood count test to measure concentration of platelets and exclude acute inflammation was used. Plasma PF4 levels were determined by ELISA.

## Results

Higher levels of PF4 (mean value - 13 pg/mL) observed in plasma of early stage NSCLC patients compared to advanced stage NSCLC (mean value - 10,5 pg/mL) and healthy individuals (mean value - 10,6 pg/mL) (p<0.02). Thrombocyte level and thrombocytosis found in patients with advanced NSCLC did not correlate with PF4 levels (p<0.01).

## Conclusion

Many cancers have a complex chemokine network. Plasma chemokines as PF4 are significant in developing theories concerning the biology of NSCLC and are potential key molecules for early detection and treatment of cancer.

